# Prefeeding of *Clarias gariepinus* with *Spirulina platensis* counteracts petroleum hydrocarbons-induced hepato- and nephrotoxicity

**DOI:** 10.1038/s41598-024-57420-4

**Published:** 2024-03-27

**Authors:** Alaa El-Din H. Sayed, Nasser S. Abou Khalil, Alshaimaa A. I. Alghriany, Sary Kh. Abdel-Ghaffar, Asmaa A. A. Hussein

**Affiliations:** 1 Molecular Biology Research and Studies Institute, Assiut University, Assiut, 71516 Egypt; 2 Zoology Department, Faculty of Science, Assiut University, Assiut, 71516 Egypt; 3 Department of Medical Physiology, Faculty of Medicine, Assuit University, Assiut, 71516 Egypt; 4 Department of Basic Medical Sciences, Faculty of Physical Therapy, Merit University, Sohag, Egypt; 5 Department of Pathology and Clinical Pathology, Faculty of Veterinary of Medicine, Assiut University, Assiut, 71516 Egypt; 6 School of Veterinary Medicine, Badr University, Assiut, Egypt

**Keywords:** Petroleum hydrocarbons, Fish, Microalga, Liver, Kidney, Biochemistry, Zoology

## Abstract

Petroleum aromatic hydrocarbons are considered one of the most dangerous aquatic pollutants due to their widespread across water bodies, persistence, and extension to the food chain. To our knowledge, there hasn’t been any research investigating the hepatorenoprotective effects of *Spirulina platensis* (SP) against toxicity induced by these environmental toxicants in fish. Thus, we decided to explore its potential safeguarding against benzene and toluene exposure in adult *Clarias gariepinus*. To achieve this objective, fish were divided into five groups (60 per group; 20 per replicate). The first group served as a control. The second and third groups were intoxicated with benzene and toluene at doses of 0.762 and 26.614 ng/L, respectively for 15 days. The fourth and fifth groups (SP + benzene and SP + toluene, respectively) were challenged with benzene and toluene as previously mentioned following dietary inclusion of SP at a dose of 5 g/kg diet for 30 days. The marked increase in liver metabolizing enzymes, glucose, total protein, albumin, globulin, albumin/globulin ratio, and creatinine confirmed the hepato- and nephrotoxic impacts of benzene and toluene. These outcomes were coupled with cytopathological affections and excessive collagen deposition. The incorporation of SP in ration formulation, on the contrary, restored the previously mentioned toxicological profile due to its antioxidant and cytoprotective attributes. Regardless of SP intervention, the renal tissues still displayed histo-architectural lesions, because of insufficient dose and timeframe. Additional research will be required to identify the ideal SP remediation regimen.

## Introduction

Studying the impact of environmental pollutants caused by human activities on the physiology of hardy fish species, is a significant concern within the realm of aquaculture. Previous studies have focused on *Heteropneustes fossilis*, the Asian stinging fish, showcasing its exceptional resilience in adverse conditions. It can endure extended periods without water for up to 72 h and survive for months within mudflats during natural and commercial scenarios. Its unique respiratory organ allows efficient oxygen extraction during dehydration stress. Additionally, the fish displays remarkable adaptability, thriving in salinities ranging from as low as 6 ppt to as high as 9 ppt, showcasing its ability to endure and adapt across a wide salinity spectrum^1,2^.

In the Egyptian environment, African catfish (*Clarias gariepinus*) holds the second position within the aquaculture sector^3^. Its omnivorous consumption pattern and preference for depth-dwelling prey are significant attributes to consider when monitoring hazardous chemicals that build up in bottom debris^4^. It is frequently employed as a model in petrochemical pollution studies^5–8,9^ due to several reasons. Firstly, it exhibits a broad tolerance range and adaptability to various environmental conditions, making it suitable for studying the impacts of pollutants in diverse aquatic habitats^10,11^. Secondly, its hardiness and ease of maintenance in controlled laboratory settings facilitate long-term exposure studies^12^. Additionally, the species’ physiological, biochemical, and histological characteristics are well-documented^13^, providing researchers with a comprehensive understanding of its responses to petrochemical pollutants. Lastly, its economic significance in aquaculture^14^ also makes it a valuable model for understanding the potential effects of petrochemical contamination on commercially important fish species.

The accelerated civilizational race is associated with contamination of water bodies with a variety of pollutants including aromatic monocyclic hydrocarbon compounds, especially benzene and toluene^15^. The latter compounds are used on a wide scale in petrochemical industries and household activities as chemical intermediates, solvents, fuel additives, and cleaners^16^. They have access to aquatic ecosystems through natural seeps, atmospheric deposition, urban runoff, sewage disposal, coastal refineries, transport losses, and combustion of fossil fuel^17,18^. In rural regions, the estimated benzene concentration is 0.06 parts per billion (ppb), but in densely populated areas and industrial zones, it can escalate to 107 ppb. Elevated benzene levels, reaching up to 3000 ppb, have been observed in the air surrounding petrol stations. Toluene averages between 1.3 and 6.6 ppb in suburban atmospheres^19^, yet areas with heavy traffic show detected toluene levels reaching 350 ppb^20^. Their presence poses serious safety concerns not only to aquatic creatures but also can be extended to consumers through the food chain due to their hydrophobicity^21^. Alterations in the gene encoding proteins which are implicated in postnatal hepatocyte differentiation and regulation of glucose and lipid homeostasis in the liver were found following exposure of *Danio rerio* embryo to benzene^15^. The cultivation of *Clarias gariepinus* in water contaminated with benzene^22^ or gradual doses of toluene^23^ resulted in a marked rise in the liver functional enzymes suggesting disruption of membrane integrity and tissue dysfunction. Benzene and Toluene increased lipid peroxidation derivatives and decreased enzymatic antioxidant activities in the liver of juvenile stages of *Clarias gariepinus*^6^. A marked change was observed in the glutathione redox system and superoxide dismutase in the liver and kidney of *Astyanax altiparanae* when challenged with a complex mixture of monocyclic aromatic hydrocarbons^24^ Engine oil compromised the cyto-biological features in the kidney of *Oreochromis niloticus* by depleting reduced glutathione and superoxide dismutase leading to the accumulation of proteinoid metabolic waste products along with adverse modifications on the characteristics of the nephron, melanomacrophage center, and microvasculature^25^.

*Spirulina platenesis* (SP), a photosynthetic microalga, succeeded in mitigating the chemotoxicants-induced hepato- and nephro-toxicities through restoring the redox balance, functional parameters, and cyto- architecture and blocking lipid peroxidation cascade in *Clarias gariepinus* intoxicated with chlorpyrifos^26^, and gibberellic acid^27^, sodium sulfate^28^, copper sulfate and copper oxide nanoparticles^29^. SP is renowned for its rich antioxidant and cytoprotective compounds such as phenolic compounds, carotenoids, vitamins, and pigments^30^ holds potential for mitigating health risks associated with petroleum hydrocarbons. Therefore, this study hypothesizes that SP supplementation will demonstrate a protective effect against benzene and toluene-induced hepato-nephrotoxicity in adult *Clarias gariepinus*. It should be noted that there is no available data on the protective effects of SP against toxicity induced by monocyclic aromatic hydrocarbons.

## Materials and methods

### Chemicals and microalgae

HPLC benzene and toluene were received from (Sigma-aldrich, Cairo, Egypt). SP 100% was purchased from Japan Algae Company (Tokyo, Japan). The nutritional composition of 100 g of SP was described in the product package (Lot number 3009). The nutritional constituents of SP were recorded in our previous investigation^31^. It comprises of total protein (43.4 ± 3.13%), total carbohydrate (28.1 ± 2.2%), total lipid (5.02 ± 1.7%), α-chlorophyll (0.68 ± 0.02%), carotenoids (0.65 ± 0.00%), and phycobiliproteins (18.43 ± 0.24%).

### Experimental protocol

The fish (*Clarias gariepinus*, weight 300–350 g, length 26–29 cm) were collected from aquaponics of the Fish Biology and Pollution Laboratory, Faculty of Science, Assiut University, Egypt. The fish were parasitic-free according to American Fisheries Society, Fish Health Section^32^. Fish were habituated for a month in glass aquariums had dimensions of 100 cm × 70 cm × 50 cm. They were subjected to rearing water which had standard physicochemical characteristics (conductivity 260.8 mM/cm, pH 7.4, dissolved oxygen 6.9 mg/L, temperature 20.5 °C, and photoperiod 12:12 h light: dark) Throughout the adaptation stage, fish received a commercially available feed contains 30% protein. In concomitant with re-dosing, 50% of the water was renewed daily.

Following the adaption phase, fish were allocated randomly into five distinct categories. Each category had 60 samples (20 fish per replicate) in glass containers with a fixed volume of water (100 L). The first group was not supplemented with any intervention except a control diet in purified water. The aquariums of the second and third groups were polluted with benzene and toluene at sublethal doses of 0.762 and 26.614 ng/L, respectively for 15 days. The quantities of benzene and toluene administered were determined through field studies conducted to investigate residues of commonly used products along the Nile River in four cities within the Assiut governorate^9^. The rationale behind choosing these doses was their capacity to induce disturbances in the redox equilibrium and trigger histopathological alterations in the liver and kidneys Sayed et al.^7^. The fourth and fifth groups (SP + benzene and SP + toluene, respectively) were intoxicated with benzene and toluene as previously mentioned following the dietary addition of SP at a dose of 5 g/kg diet for 30 days. The reason for selecting this specific dose and duration of exposure was their capability to mitigate hepatic injury by reinstating hepatic function markers and balancing the oxidant and antioxidant status based on a dose-dependent study^33^.

### Sample collection

Six fish from each group were gathered at the end of the experimental period and anesthetized with ice. Blood samples had been obtained from the caudal veins and left to clot in plain centrifuge tubes at room temperature before being centrifuged at 5000 rpm for 20 min under cooling to separate serums for the subsequent biochemical examination.

Small pieces of livers and kidneys were promptly preserved in 10% neutral buffered formalin (pH 7.2). Using the paraffin-embedding technique, these selected pieces were routinely processed. Then, they were washed, dehydrated in ethanol solutions of escalating concentrations (from 70 to 100%), and cleaned in xylene before being embedded in wax. Using a rotatory microtome, paraffin sections were sliced to a thickness of 5 µm, and then the paraffin was removed with xylene. For a general histological study, standard Hematoxylin and Eosin stains were used, and the Masson trichrome stain was used for identifying collagen fibers^34^. Examination and photography were done with a digital camera (Toup Tek ToupView, Copyright© 2019, Version: × 86, Compatible: Windows XP/Vista/7/8/10, China), ImageJ software, and a computer linked to a light microscope (Olympus CX31, Japan).

### Biochemical measurements

Aspartate aminotransferase (AST), alanine aminotransferase (ALT), alkaline phosphatase (ALP), glucose, total protein (TP), albumin, and creatinine were measured by commercial colorimetric kits following the manufacturer’s instructions (SGMitalia Company, USA). The analysis protocols for AST, ALT, ALP, glucose, and TP involved measuring sample absorbances against the reagent blank at 546 nm. For creatinine, absorbances of standards and samples were measured at 492 nm. In albumin estimation, sample and standard absorbances were measured against the reagent blank at 623 nm. Globulin was calculated by subtracting albumin values from TP. The albumin/globulin ratio (A/G ratio) was determined by dividing albumin values by globulin values.

Evaluation of total antioxidant capacity (TAC) was based on the protocol of a colorimetric kit (Biodiagnostic, Egypt, catalog number: TA2513). According to this method, the absorbances of blank and samples were measured against distilled water at 500 nm. Catalase (CAT) was determined using 3,5-dichloro-2-hydroxybenzene sulfonic acid to rapidly end the degradation reaction of hydrogen peroxide catalyzed by CAT, generating a yellow reaction product with residual hydrogen peroxide^35^. The absorbances of samples were recorded against sample blank, and standard against standard blank at 510 nm. Superoxide dismutase (SOD) was assessed based on its ability to inhibit the phenazine methosulphate-mediated reduction of nitroblue tetrazolium dye to form a red product^36^. The reaction was run at 30 °C in spectrophotometer at 480 nm for 3 min. Malondialdehyde (MDA) levels were measured using tetramethoxypropane as an external standard^37^. The absorbances were measured at 532 nm against n-butanol/pyridine as a blank. Total peroxide (TPX) was assessed following the protocol of^38^ and calculated from the standard curve constructed using a series of standard concentrations. TPX content of the plasma samples was determined as a function of the difference in absorbance between the test and blank samples using a solution of H2O2 as standard at 560 nm. Oxidative stress index (OSI), as a percentage ratio of TPX content to TAC concentration, was estimated according to the following equation^38^: OSI = (TPX, μM/L)/(TAC, μM/L) × 100.

### Statistical analysis

Data were represented as mean ± standard error of the mean (SEM). The results were analyzed by one-way analysis of variance (ANOVA) followed by Duncan post-test using SPSS program version 16 (SPSS Inc., Chicago, USA). Differences of *p* < 0.05 were considered to be statistically significant.

### Ethics approval and consent to participate

Studies were approved by the Research Ethics Committee of the Molecular Biology Research and Studies Institute (MB-21-27-R), Assiut University, Assiut, Egypt. All methods were carried out following the relevant regulations and ARRIVE guidelines.

## Results

### Serum liver and kidney function parameters

As shown in Table 1, *Clarias gariepinus* challenged with each of the examined compounds were characterized by a significant rise in AST (73.43 ± 0.36 and 62.52 ± 0.88 U/L in the benzene and toluene groups, respectively), ALP (33.83 ± 0.30 and 30.90 ± 0.20 U/L in the benzene and toluene groups, respectively), glucose (130.12 ± 1.20 and 128.30 ± 1.36 mg/dL in the benzene and toluene groups, respectively), TP (6.75 ± 0.26 and 5.53 ± 0.27 g/dL in the benzene and toluene groups, respectively), albumin (1.77 ± 0.02 and 1.42 ± 0.60 g/dL in the benzene and toluene groups, respectively), and globulin (2.73 ± 0.02 and 2.48 ± 0.02 g/dL in the benzene and toluene groups, respectively), compared to the control group (AST activity was 60.48 ± 0.41 U/L, ALP activity was 27.55 ± 0.23 U/L, glucose level was 112.27 ± 0.91 mg/dL, TP level was 4.80 ± 0.07 g/dL, albumin level was 1.22 ± 0.02 g/dL, and globulin level was 2.32 ± 0.02 g/dL) indicating liver dysfunction. In the benzene group, AST increased by 21.41%, while in the toluene group, it rose by 3.37%. In terms of ALP, there was a rise of 22.79% in the benzene group and 12.16% in the toluene group. Glucose levels increased by 15.90% and 14.28% in the benzene and toluene groups, respectively. TP exhibited a 40.63% increase in the benzene group and 15.21% in the toluene group. Additionally, albumin increased by 45.10% and 16.39% in the benzene and toluene groups, while globulin rose by 17.67% in the benzene group and 6.90% in the toluene group. Intoxication with benzene was responsible for a significant rise in ALT (35.53 ± 0.38 U/L) and creatinine (0.88 ± 0.02 mg/dL) versus the control group (ALT activity was 31.45 ± 0.38 U/L, and creatinine level was 0.62 ± 0.02 mg/dL). In the benzene group, ALT and creatinine increased by 12.975% and 41.94%, respectively. The A/G ratio in benzene and toluene intoxicated groups (0.64 ± 0.01 and 0.57 ± 0.02, respectively) was significantly higher than that in the control one (0.52 ± 0.01). The A/G ratio increased by 23.08% in the benzene group and 9.62% in the toluene group. AST (69.95 ± 0.35 U/L), ALT (33.83 ± 0.35 U/L), ALP (30.83 ± 0.33 U/L), glucose (123.92 ± 1.89 mg/dL), globulin (2.62 ± 0.03 g/dL), and creatinine (0.82 ± 0.02 mg/dL) in the SP + benzene group were significantly lower than those in the benzene group, while still significantly higher than those in the control group. In comparison with the benzene group, AST, ALT, and ALP decreased by 4.74%, 4.78%, and 8.87%, respectively, in the SP + benzene group. In the SP + benzene group compared to the benzene group, glucose decreased by 4.76%, globulin decreased by 4.03%, and creatinine decreased by 6.82%. The protective intervention with SP was highly effective in reducing the hepatic metabolizing enzymes (AST activity was 59.57 ± 0.83 U/L, ALT activity was 30.67 ± 0.32 U/L, and ALP activity was 28.25 ± 0.09 U/L) and globulin (2.38 ± 0.02 g/dL) in toluene-intoxicated *Clarias gariepinus* to restore the control level. In the SP + toluene group, AST decreased by 4.72%, ALT by 4.81%, and ALP by 8.58% compared to the toluene group. Glucose in the SP + toluene group (122.18 ± 1.30 mg/dL) was significantly lower than that in the toluene group but still higher than that in the control group. Supplementing with SP led to a 4.77% decrease in glucose level compared to the toluene group.

**Table 1:** Serum hepatic and renal function parameters in *Clarias gariepinus* intoxicated with benzene and toluene, and those prefeed with *Spirulina platensis.*

Group parameter	Control	Benzene	Toluene	SP + benzene	SP + toluene
AST activity (U/L)	60.48 ± 0.41	73.43 ± 0.36^a^	62.52 ± 0.88^a^	69.95 ± 0.35^a^^,b^	59.57 ± 0.83^c^
ALT activity (U/L)	31.45 ± 0.38	35.53 ± 0.38^a^	32.22 ± 0.34	33.83 ± 0.35^a^^,b^	30.67 ± 0.32^c^
ALP activity (U/L)	27.55 ± 0.23	33.83 ± 0.30^a^	30.90 ± 0.20^a^	30.83 ± 0.33^a^^,b^	28.25 ± 0.09^c^
Glucose level (mg/dL)	112.27 ± 0.91	130.12 ± 1.20^a^	128.30 ± 1.36^a^	123.92 ± 1.89^a^^,b^	122.18 ± 1.30^a^^,^^c^
TP level (g/dL)	4.80 ± 0.07	6.75 ± 0.26^a^	5.53 ± 0.27^a^	6.43 ± 0.23^a^	5.28 ± 0.25^a^
Albumin level (g/dL)	1.22 ± 0.02	1.77 ± 0.02^a^	1.42 ± 0.60^a^	1.67 ± 0.02^a^	1.38 ± 0.04^a^
Globulin level (g/dL)	2.32 ± 0.02	2.73 ± 0.02^a^	2.48 ± 0.02^a^	2.62 ± 0.03^a^^,b^	2.38 ± 0.02^c^
A/G ratio	0.52 ± 0.01	0.64 ± 0.01^a^	0.57 ± 0.02^a^	0.64 ± 0.01^a^	0.57 ± 0.02^a^
Creatinine level (mg/dL)	0.62 ± 0.02	0.88 ± 0.02^a^	0.67 ± 0.03	0.82 ± 0.02^a^^,b^	0.65 ± 0.02

Results are expressed as the mean ± SEM of 6 fish per group (One-way ANOVA followed by Duncan post-test).

*SP* *Spirulina platensis*, *AST* aspartate aminotransferase, *ALT* alanine transaminase, *ALP* alkaline phosphatase, *TP* total protein, *A/G ratio* albumin/globulin ratio.

^a^Significant difference between the control group and any of the other treated groups at *p* < 0.05.

^b^Significant difference between benzene group and SP + benzene group at *p* < 0.05.

^c^Significant difference between toluene group and SP + toulene group at *p* < 0.05.

### Serum redox parameters

The disturbance in oxidant/antioxidant balance following the exposure to monocyclic hydrocarbon chemicals was manifested by a significant elevation in MDA (4.86 ± 0.01 and 4.63 ± 0.01 nmol/mL in the benzene and toluene groups, respectively), TPX (2.11 ± 0.03 and 1.90 ± 0.05 μM/L in the benzene and toluene groups, respectively), TAC (1.21 ± 0.01 and 1.18 ± 0.00 μM/L in the benzene and toluene groups, respectively), OSI (174.13 ± 1.59 and 161.58 ± 3.90% in the benzene and toluene groups, respectively), SOD (13.76 ± 0.06 and 13.49 ± 0.04 U/L in the benzene and toluene groups, respectively), and CAT (12.56 ± 0.03 and 12.45 ± 0.03 U/L in the benzene and toluene groups, respectively) relative to the control group (MDA level was 4.13 ± 0.01 nmol/mL, TPX was 1.67 ± 0.04 μM/L, TAC was 1.06 ± 0.00 μM/L, OSI was 157.92 ± 1.94%, SOD activity was 11.80 ± 0.04 U/L, and CAT activity was 10.86 ± 0.03 U/L). The MDA levels rose by 17.68% in the benzene group and 12.11% in the toluene group compared to the control. TPX increased by 26.35% in the benzene group and 13.77% in the toluene group. TAC showed a rise of 14.15% in the benzene group and 11.32% in the toluene group. OSI increased by 10.26% in the benzene group and 2.32% in the toluene group. Additionally, SOD increased by 16.61% in the benzene group and 14.32% in the toluene group, while CAT increased by 15.65% in the benzene group and 14.64% in the toluene group, all in comparison to the control group. All the redox biomarkers in *Clarias gariepinus* exposed to the studied gasoline components were significantly reduced by pre-feeding with SP, but they were still significantly higher (MDA level was 4.42 ± 0.01 and 4.21 ± 0.01 nmol/mL, TAC was 1.10 ± 0.01 and 1.07 ± 0.00 μM/L, OSI was 174.13 ± 1.59 and 161.58 ± 3.90%, SOD activity was 12.51 ± 0.05 and 12.27 ± 0.04 U/L, and CAT activity was 11.42 ± 0.03 and 11.32 ± 0.03 U/L in the SP + benzene and SP + toluene groups, respectively) than the control group. The SP + benzene group showed a 9.05% decrease in MDA compared to the benzene group. TAC, SOD, and CAT also exhibited declines of 9.09%, 9.08%, and 9.08%, respectively, in the SP + benzene group versus the benzene group. Following the intervention of SP in toluene-intoxicated fish, MDA reduced by 9.07%, TAC decreased by 9.32%, SOD decreased by 9.04%, and CAT decreased by 9.08%. The exception to this outcome pattern is the TPX of the SP + toluene group (1.73 ± 0.04 μM/L) which successfully reduced to the control level (Table 2). The percentage of reduction in TPX was 8.95% in the SP + toluene group compared to the toluene group.

**Table 2:** Serum redox parameters in *Clarias gariepinus* intoxicated with benzene and toluene, and those prefeed with *Spirulina platensis.*

Group parameter	Control	Benzene	Toluene	SP + benzene	SP + toluene
MDA level (nmol/mL)	4.13 ± 0.01	4.86 ± 0.01^a^	4.63 ± 0.01^a^	4.42 ± 0.01^a^^,^^b^	4.21 ± 0.01^a^^,c^
TPX level (μM/L)	1.67 ± 0.04	2.11 ± 0.03^a^	1.90 ± 0.05^a^	1.91 ± 0.02^a^^,^^b^	1.73 ± 0.04^c^
TAC (μM/L)	1.06 ± 0.00	1.21 ± 0.01^a^	1.18 ± 0.00^a^	1.10 ± 0.01^a^^,^^b^	1.07 ± 0.00^a^^,c^
OSI (%)	157.92 ± 1.94	174.13 ± 1.59^a^	161.58 ± 3.90^a^	174.13 ± 1.59^a^	161.58 ± 3.90^a^
SOD activity (U/L)	11.80 ± 0.04	13.76 ± 0.06^a^	13.49 ± 0.04^a^	12.51 ± 0.05^a^^,^^b^	12.27 ± 0.04^a^^,c^
CAT activity (U/L)	10.86 ± 0.03	12.56 ± 0.03^a^	12.45 ± 0.03^a^	11.42 ± 0.03^a^^,^^b^	11.32 ± 0.03^a^^,^^c^

Results are expressed as the mean ± SEM of 6 fish per group (One-way ANOVA followed by Duncan post-test).

*SP* *Spirulina platensis*, *MDA* malondialdehyde, *TPX* total peroxide, *TAC* total antioxidant capacity, *OSI* oxidative stress index, *SOD* superoxide dismutasem, *CAT* catalase.

^a^Significant difference between the control group and any of the other treated groups at *p* < 0.05.

^b^Significant difference between benzene group and SP + benzene group at *p* < 0.05.

^c^Significant difference between toluene group and SP + toulene group at *p* < 0.05.

### Histopathological findings

**Figure 1:**
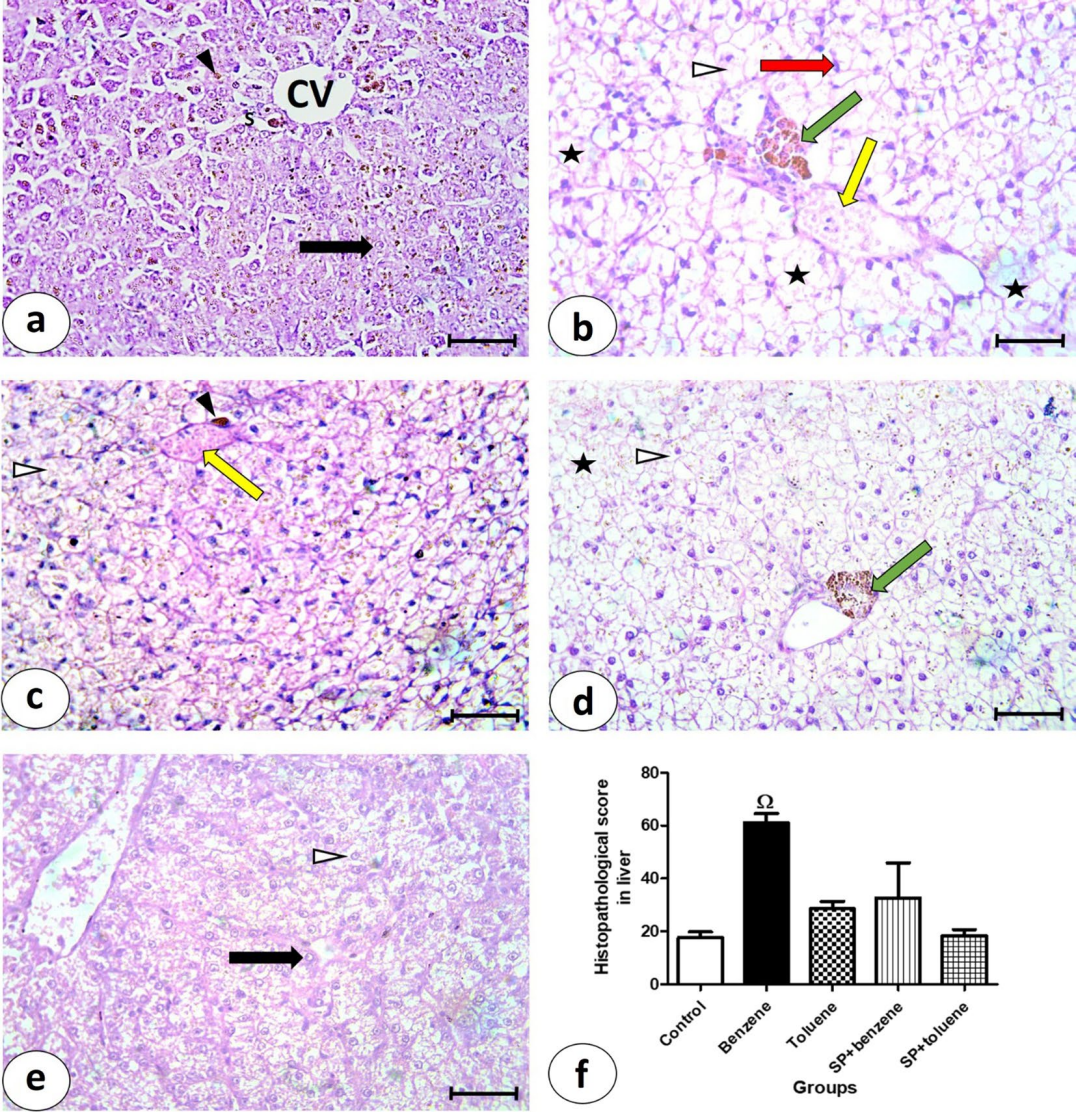
Photomicrographs of liver sections stained by H&E, bars = 50 μm (**a**–**e**). (**a**) Control group showing central vein (CV), hepatocytes with rounded vesicular nuclei (black arrow), blood sinusoids (S), and pigments (▲). (**b**) Benzene group showing vacuolated hepatocytes (∆), with dense eccentric nuclei (red arrow), congested portal vein (yellow arrow), necrotic areas (asterisk), and melanomachrophge cells in the portal area (green arrow). (**c**) Toluene group showing some vacuolated hepatocytes (∆), congested dilated blood sinusoid (yellow arrow), and melanomachrophge cells (▲). (**d**) SP + Benzene group showing vacuolated hepatocytes (∆), necrotic areas (asterisk), and melanomachrophge cells in the portal area (green arrow). (**e**) In SP + Toluene group showing normal hepatocytes with rounded vesicular nuclei and acidophilic cytoplasm (black arrow), and few vacuolated cells (∆). (**f**) Histopathological score in liver tissue from the experimental groups.

Liver sections stained by hematoxylin and eosin from the control group revealed the typical structure of the liver (Fig. 1a). Hepatocytes were radiated from central veins in cords separated by blood sinusoids. The hepatocytes had centric or eccentric rounded vesicular nuclei and prominent nucleoli. Melanomacrophage cells were observed in the hepatic tissue. The liver from the benzene group (Fig. 1b) showed vacuolated hepatocytes with dense eccentric nuclei, congested portal veins, necrotic areas, and pigments in the portal area. The toluene group (Fig. 1c) showed some vacuolated hepatocytes, congested dilated blood sinusoids, and melanomacrophage cells. The SP + benzene group (Fig. 1d) showed vacuolated hepatocytes, necrotic areas, and melanomacrophage cells in the portal area. In the SP + toluene group (Fig. 1e), the hepatocytes were normal and had rounded vesicular nuclei and acidophilic cytoplasm. Few cells were vacuolated. The histopathological score of five degenerative parameters (vacuolation of hepatocytes, pyknosis, necrotic areas, cellular infiltrations, and congestion of blood vessels) was done for all the experimental groups (Fig. 1f). The histopathological score from benzene group (61.00 ± 3.51) increased significantly when compared with control group (17.67 ± 2.19) while, SP in SP + benzene group (32.67 ± 13.25) decreased it significantly versus benzene group. The hepatic histopathological score showed a 245.23% increase in the group exposed to benzene compared to the control group. However, intervention with SP in benzene-intoxicated fish led to a reduction of 46.44% in the hepatic histopathological score compared to the benzene-exposed group.

**Figure 2:**
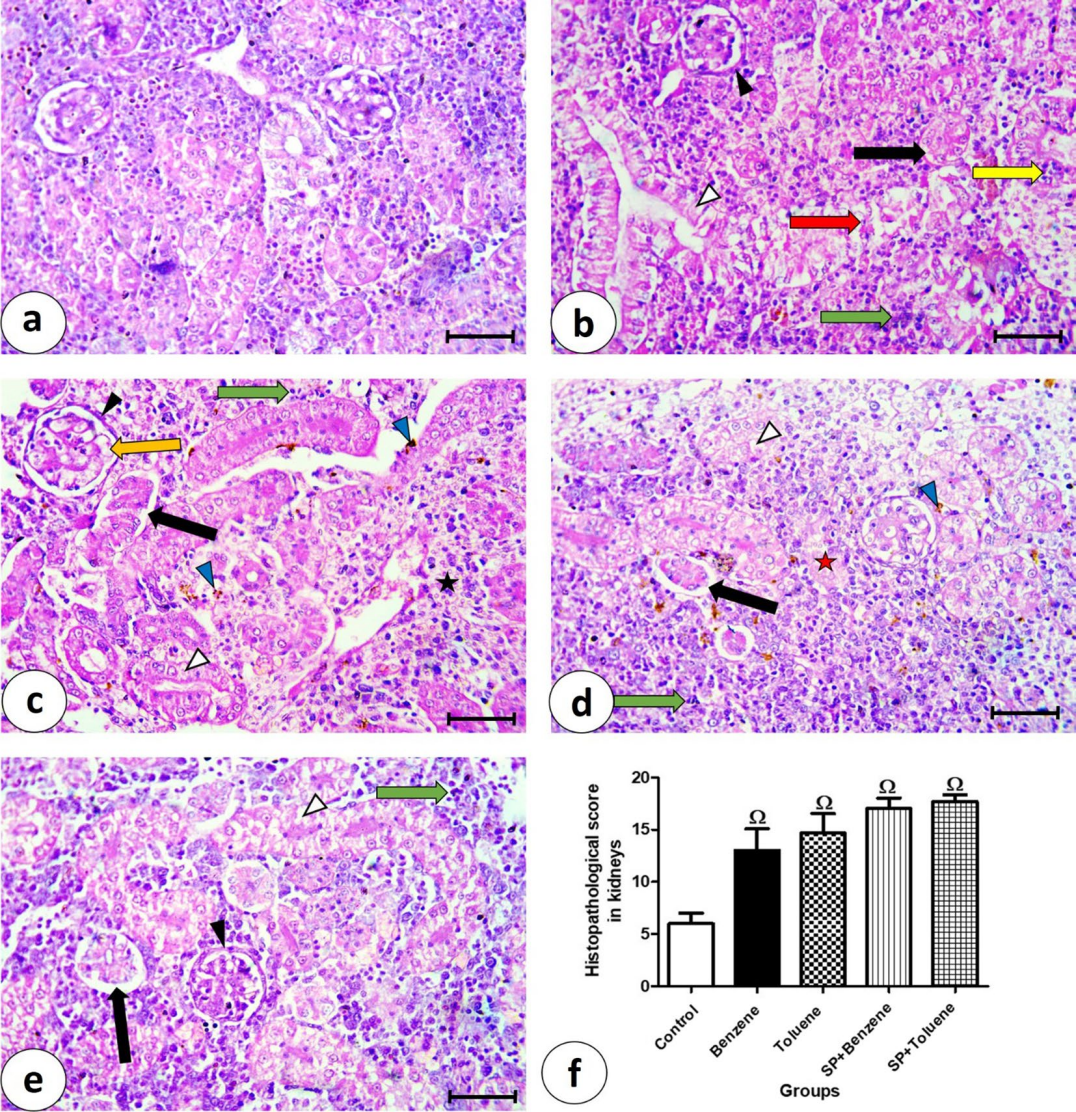
Photomicrographs of kidney sections stained by H&E, bars = 50 μm (**a**–**e**). (**a**) Control group showing the normal renal tissue. (**b**) Benzene group showing thickening in the Bowman’s capsule (▲), epithelial separation of renal tubules (black arrow), renal tubules with vacuolation (∆), desquamation (red arrow), and cellular infiltration in renal tissue (green arrow). (**c**) In Toluene group showing thickening in the Bowman’s capsule (▲), vacuolation in the glomeruli (orange arrow), epithelial separation of renal tubules (black arrow), vacuolated renal tubular cells (∆), cellular infiltration (green arrow), hemorrhage around injured blood vessel (asterisk), and cells containing pigments (blue arrowhead). (**d**) SP + Benzene group showing epithelial separation of renal tubules (black arrow), cellular infiltration (green arrow), necrotic areas (red asterisk), vacuolated renal tubular cells (∆), and large number of cells containing pigments (blue arrowhead). (**e**) In SP + Toluene group thickening in the Bowman’s capsule (▲), epithelial separation of renal tubules (black arrow), vacuolated renal tubular cells (∆), and cellular infiltration (green arrow). (**f**) Histopathological score in renal tissue from the experimental groups.

Kidney sections from the control group showed the typical renal tissue structure (Fig. 2a). The benzene group (Fig. 2b) showed thickening in the Bowman’s capsule, epithelial separation of renal tubules. Renal tubular epithelium showed vacuolation and desquamation. Cellular infiltration in renal tissue was observed. The toluene group (Fig. 2c) showed thickening in the Bowman’s capsule, vacuolation in the glomeruli, epithelial separation of renal tubules, vacuolated renal tubular cells, cellular infiltration, hemorrhage around injured blood vessels, and cells containing pigments. The kidney sections from the SP + benzene group (Fig. 2d) showed epithelial separation of renal tubules, cellular infiltration, necrotic areas, vacuolated renal tubular cells, and numerous pigmented cells. The SP + toluene group (Fig. 2e) showed thickening in the Bowman’s capsule, epithelial separation of renal tubules, vacuolated renal tubular epithelium cells, and cellular infiltration. The histopathological score for five degenerative parameters (vacuolation of renal tubular cells, thickening of Baumann’s capsule, separation of epithelial lining of renal tubules, interstitial cellular infiltration, hemorrhage) was performed for all the experimental groups (Fig. 2f). The histopathological scores from benzene and toluene groups (13.00 ± 2.08 and 14.67 ± 1.86, respectively) increased significantly when compared with the control group (6.00 ± 1.00). The histopathological scores of kidneys in the benzene and toluene groups showed an increase of 116.67% and 144.50%, respectively, compared to the control group.

### Collagen fibers examination

**Figure 3:**
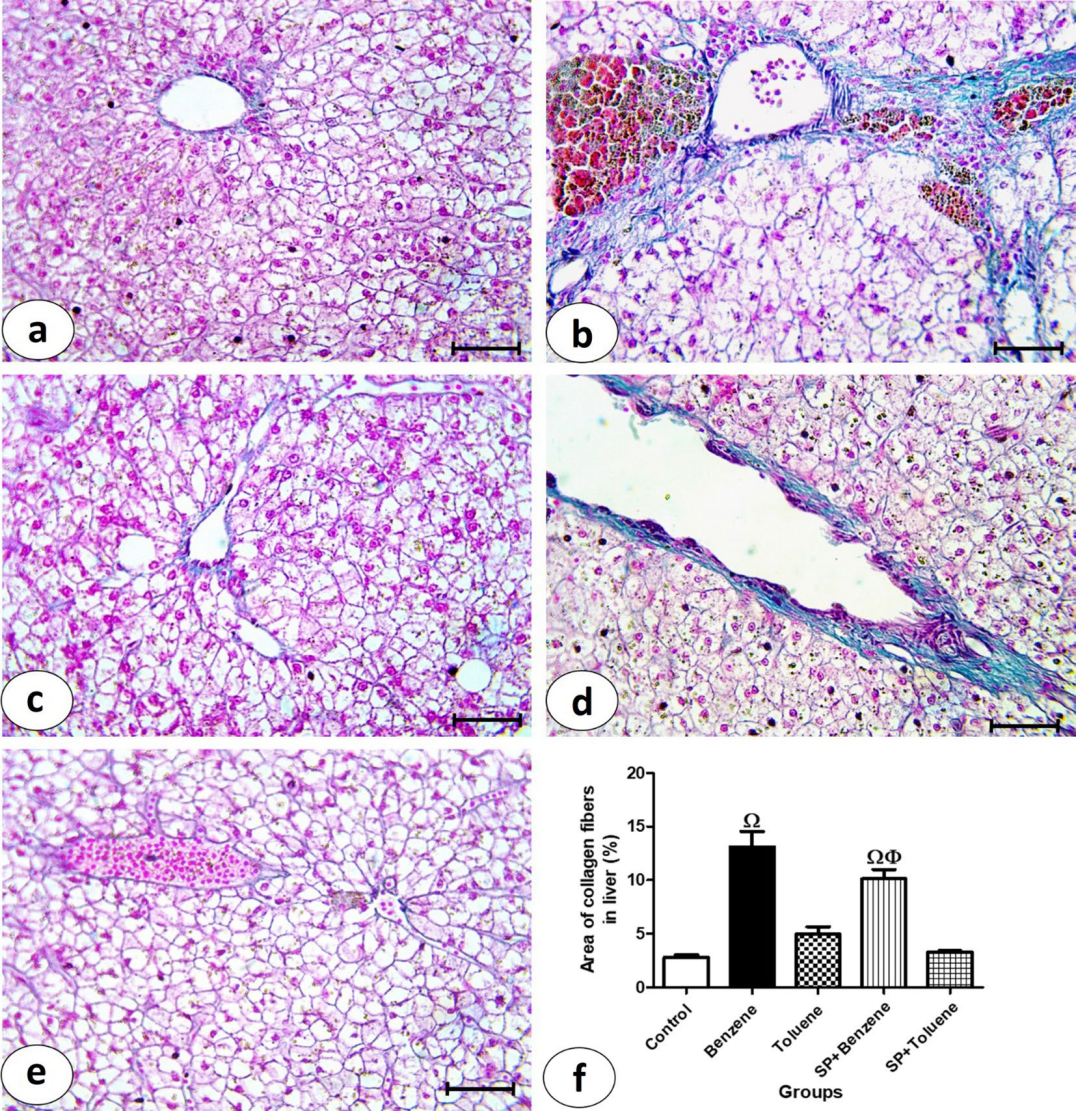
Examination of collagen fibers in liver sections from experimental animal groups. Photomicrographs of liver sections stained by Masson’s trichrome (**a**–**e**), bar = 50 μm. (**a**) Control group showing normal few amounts of collagen fibers in hepatic tissue. (**b**) In Benzene group showing great amount of collagen fibers appeared as green color. (**c**) In Toluene group showing few amounts of collagen fibers. (**d**) SP + Benzene group showing noticeable amount of collagen fibers. (**e**) In SP + Toluene group showing few amounts of collagen fibers. (**f**) Percentage of collagen fibers in liver section from all experimental groups.

Liver sections stained by Masson’s trichrome stain from the control group showed a minimal amount of collagen fibers around the central veins (Fig. 3a). Benzene group showed an excessive amount of collagen fibers around the portal area, as represented by the green color (Fig. 3b). Toluene group (Fig. 3c) showed a small amount of collagen fibers. Liver sections from the SP + benzene group (Fig. 3d) showed a noticeable amount of collagen fibers. In the SP + toluene group, there were fewer collagen fibers (Fig. 3e). The percentage of the area of collagen fibers in the benzene group (11.46 ± 0.85%) increased significantly compared to the control group (3.09 ± 0.42%) (Fig. 3f). SP in the SP + benzene group decreased the percentage (5.48 ± 1.16%) significantly versus benzene group. The increase in the percentage of the benzene group was significant compared to that of the toluene group (9.31 ± 1.20%). The benzene group exhibited a 366.19% increase in the percentage of collagen fiber area in the liver compared to the control group. In the SP + benzene group, SP intervention resulted in a 22.75% reduction in this percentage compared to the benzene group. Additionally, the percentage in the benzene group increased by 163.58% compared to the toluene group.

**Figure 4:**
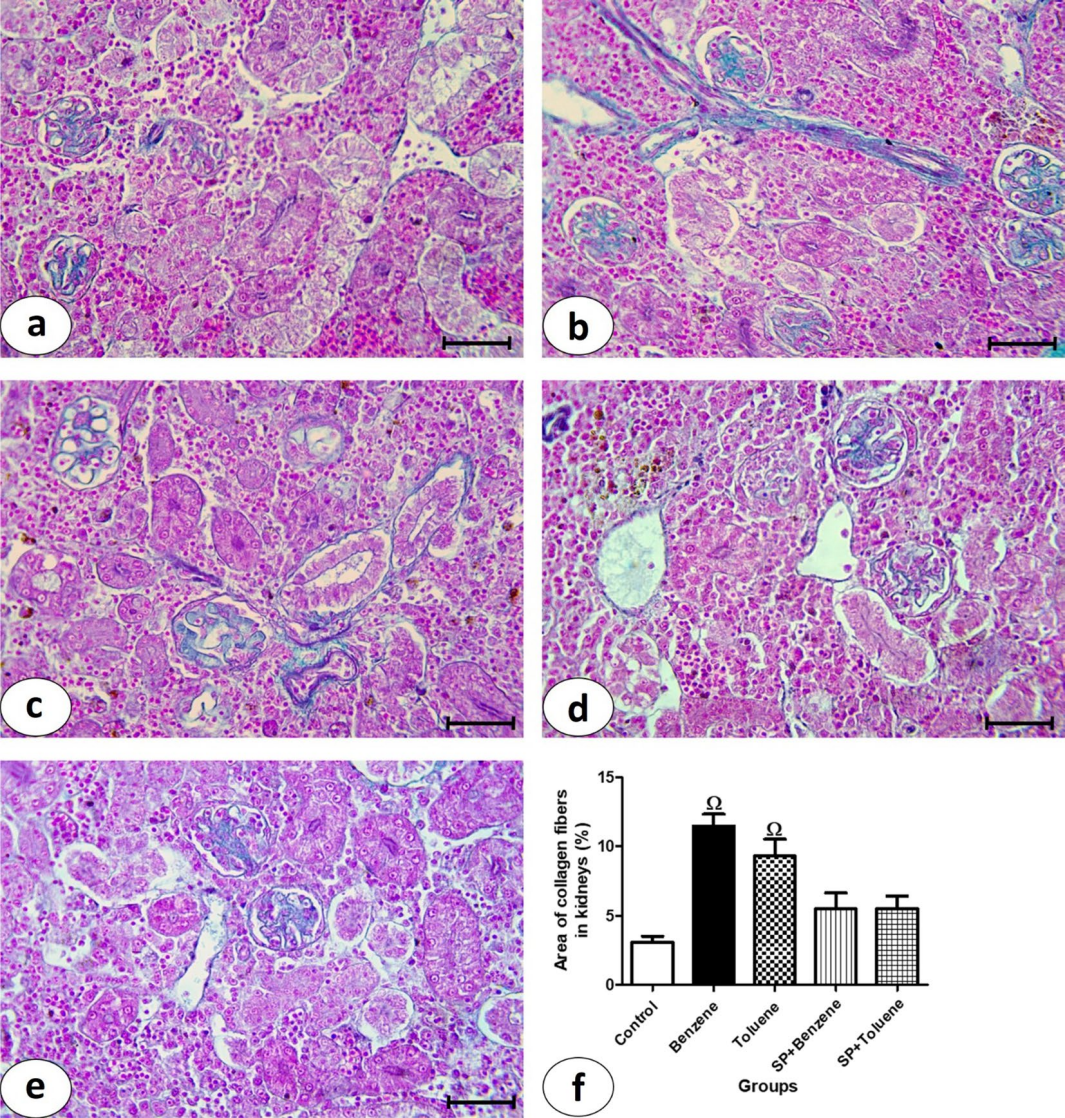
Examination of collagen fibers in kidney sections from experimental animal groups. Photomicrographs of kidney sections stained by Masson’s trichrome (**a**–**e**), bar = 50 μm (**a**) Control group showing mild amount of collagen fibers in renal tissue. (**b**) Benzene group showing marked increase of collagen fibers amount appeared as green color. (**c**) Toluene group showing noticeable amount of collagen fibers. (**d**) and (**e**) SP + Benzene group and SP + Toluene group showing moderate amount of collagen fibers. (**f**) Percentage of collagen fibers in kidney section from all experimental groups.

Collagen fibers in kidney sections stained by Masson’s trichrome stain from the control group showed a mild amount of collagen fibers in renal tissue (Fig. 4a). Benzene group (Fig. 4b) and toluene group (Fig. 4c) showed a marked increase in the amount of collagen fibers, as represented by the green color. A moderate amount of collagen fibers was observed in SP + benzene (Fig. 4d) and SP + toluene groups (Fig. 4e). Statistically, in benzene group, the percentage of the area of collagen fibers (13.10 ± 1.46%) increased significantly compared to that of the control group (2.81 ± 0.24%) (Fig. 4f). Spirulina in the SP + benzene group decreased the percentage of collagen fibers (10.12 ± 0.90%) significantly versus the benzene group. The percentage also significantly decreased in the SP + toluene group (3.28 ± 0.14%) compared to the toluene group (4.97 ± 0.70%). There was a 270.87% increase in the percentage of collagen fiber area in the kidney compared to the control group. In the SP + benzene group, the administration of spirulina resulted in a 52.18% reduction in the percentage of collagen fibers compared to the benzene group. Additionally, in the SP + toluene group, there was a 40.71% decrease in the percentage compared to the toluene group.

## Discussion

The rise in the activity of hepatic metabolizing enzymes following the exposure to hydrophobic components of gasoline corresponds to the findings in *Clarias gariepinus* cultivated in aquaria contaminated with benzene^22^. This outcome could be due to stimulation of gluconeogenesis from amino acid precursors in intoxicated fish^39^. It also could point to an interruption of intrahepatic and extrahepatic bile passage and hepatobiliary breakdown^40^.

The hyperglycemia in petroleum hydrocarbons-exposed fish is compatible with that observed in *Prochilodus lineatus* exposed to water-soluble fractions of gasoline^41^, implying a compensatory reactivity to deal with the extensive metabolic requirements and subsequent stress^39^. This secondary stress response could be due to the augmented output of cortisol mobilizing glucose from its cellular stores by activating gluconeogenesis and glycogenolysis pathways to deal with the extra energy requests^42^.

The increased plasma TP level of our intoxicated models is in parallel with *Clarias gariepinus* intoxicated with microplastics^43^ and Voliam flexi^®^^11^, but in contradiction to *Prochilodus lineatus* exposed to diesel oil, a rich source of monocyclic hydrocarbons^44^. This inconsistent data may arise from differences in the fish species and/or experimental duration. Increased albumin and globulin levels are responsible for the increase in TP, which point to hypermetabolic activity in the liver, another adaptive tool in response to the toxicity^45^. As a part of the compensatory scenario in the face of reactive damaging molecules, the rate of protein generation is accelerated to aid in the biosynthesis of antioxidants and cytoprotective agents^46^.

The raising in creatinine levels of the benzene group is in the same line as that observed in juvenile *Oreochromis niloticus* after exposure to water-soluble fractions of crude oil^47^. These observations were supported by renal histo-architecture deteriorations in the present and previous studies^25^, and indicate the ability of xenobiotics to impair the role of the kidney in getting rid of the metabolites by reducing glomerular membrane filterability^48^.

As an end product of the lipid peroxidation cascade, the accumulation of MDA denotes an attack of cellular membranes by a surplus of reactive oxidants^49^. As encouragement of the antioxidant network was obligatory to combat the negative influences of oxidative instability, the free radical overloading arouses gene expression of endogenous antioxidants driven by stimulation of oxidants-related transcription effectors and its downstream signaling transduction^50^. Based on this fact, the induction of CAT activity is a reflection of the overgeneration of hydrogen peroxide during the biotransformation of xenobiotics^51^. Close inspection of our findings verifies this fact as indicated by raising in the enzymatic antioxidants and overall antioxidant potency. Despite this, the peroxidation markers still elevated suggesting a failure of redox stabilizers in fighting the oxidizing intermediates.

The histopathological abnormalities in the liver and kidney of exposed groups are in the same line with those reported in the studies of Sayed et al.^7,8^. As a consequence of loss of antioxidant defensive shield to fight damaging free radicals and increase in death-mediated proteoses^52,53^, and following the observations of Sayed et al.^7,54^, our results revealed presence of necrotic alterations in the liver of benzene group and epithelial sloughing in the kidney of benzene and toluene groups. Loss of hepatocellular integrity is associated with the release of its metabolizing enzymes into the bloodstream^40^.

The hepato- and renal cellular vacuolation in the intoxicated groups could be attributed to a shift in the metabolic handling of fatty acids towards lipolysis at the expense of lipogenesis resulting in the deposition of triglycerides which is dissipated by organic solvents during tissue processing, producing unstained hollow areas^55,56^. This cytological feature was also seen in the nonylphenol-intoxicated *Clarias gariepinus* model^57^.

The accumulation of collagen in the kidney of the benzene and toluene groups and the liver of the benzene group is in harmony with peri-bronchiolar fibrosis in rats exposed to benzene vapors^58^. The modulation in gene expression related to collagen overproduction by benzene leads to extensive structural disorganization/remodeling that characterizes the fibrotic response^59^.

Benzene exposure resulted in more severe effects compared to toluene, as evidenced by significantly elevated levels of various oxidative stress markers, a significantly higher hepatic histopathological score, and a more significant increase in the percentage of collagen fibers in both the liver and kidneys. Toluene, on the other hand, showed fewer adverse effects, with no significant differences in histopathological scores and a less pronounced increase in collagen fiber percentage compared to the control group. Overall, benzene seems to exert a more adverse influence on the observed parameters compared to toluene. Earlier research have illustrated the more detrimental impact of benzene compared to toluene on the physiological and histological aspects of fish. A study on Nile tilapia (*Oreochromis niloticus*) revealed that benzene exhibited greater hepatotoxicity and nephrotoxicity than toluene and xylene, as assessed through haemato-biochemical, mutagenic, and histopathological analyses. The levels of antioxidant enzymes in the liver were higher in fish treated with benzene as opposed to those treated with toluene and xylene. The heightened toxicity of benzene attributed to its elevated vapor pressure^8^. Another investigation explored the response of Clarias gariepinus to exposure to monoaromatic petroleum hydrocarbons, utilizing oxidative stress, histopathological changes, and immunological alterations as biomarkers. The study identified benzene as the most toxic compound, followed by toluene and xylene. These findings were attributed to the increased stability and longer lifespan of benzene, making it more accessible to exposed animals compared to the other components^8^.

The reduction in the hepatic metabolizing enzymes in the petroleum hydrocarbons-challenged fish pre-fed with SP is corresponding to other intoxicated experimental models^27,29^. This finding is attributed to a diversity of phytochemicals that block the overgeneration of reactive oxidant derivatives maintaining the membrane permeability and preventing the escape of hepatic cytopathic markers into the extracellular space^33^.

The dietary inclusion of SP before intoxication with petroleum derivatives similarly restored the blood glucose balance to that found in nanocomposites-intoxicated *Oreochromis niloticus*^29^, and chlorpyrifos-intoxicated *Clarias gariepinus*^26^. Activation of hexokinase and inhibition of glucose-6-phosphatase, enhancement of beta-cell functions, tissue insulin sensitivity, and hepatic glycogenesis^60^ are responsible for the hypoglycemic action of SP.

The findings of other scientists^26,27, 29^ corroborate the clear reduction in the creatinine levels in the SP + benzene group compared to its respective exposed groups. This outcome could be attributed to the reversal of filtration barrier damage^61^ and enhancement of the vascular functionality^62^.

The reduction in the serum MDA concentration of aromatic hydrocarbons-intoxicated fish pre-feed with SP is consistent with other scholarly articles^26^. The biologically active ingredients in SP neutralize the reactive radicals, suppress NADPH oxidase (a superoxide-generating enzyme), elaborate the oxidants buffering system, and augment glutathione manufacture^63^. These characteristics may be linked to its abundance of antioxidant elements such as C-phycocyanin, β-carotene, minerals, vitamins, proteins, carbohydrates, and fat. We hypothesized that the protective time window provided by SP before contamination with hydrophobic components of gasoline enhanced the endogenous antioxidant firewall confirmed by the normalization of the antioxidant network at the end of the intervention schedule in our study and up-regulation of the enzymatic antioxidant branch in another one^64^, leading finally to the recovery of the redox status.

The histopathological improvements in the petroleum hydrocarbons-compromised liver of *Clarias gariepinus* prefeed with SP are corresponding to Sayed et al.^29,43^. These findings could be attributed to the ability of SP to retain the hepatic cytoarchitecture stability after eliminating the harmful radicals by its phytochemical antioxidant and anti-inflammatory components^33^. On the contrary, the unsuccessful attempts of SP to antagonize the histological perturbations in the kidney of challenged groups could be due to insufficient intervention duration or the supplemented dose of SP.

The anti-fibrotic activity of SP is matched with the findings in mice suffering from Western diet-induced non-alcoholic steatohepatitis^65^. This response could be due to reduced expression of peroxisome proliferator-activated receptor-alpha^66^ which plays a main role in limiting fibrosis. SP supplementation also reduces hepatic stellate cell activation, a key step in the initiation and progression of collagen deposition^67^. The anti-inflammatory^68^ and free radical scavenging properties^63^ of SP increase the turnover of the extracellular matrix and decrease its production resulting in the suppression of fibrogenesis.

## Conclusion

Hepato- and nephrotoxicity was observed following exposure of *Clarias gariepinus* to benzene and toluene as represented by raise in liver metabolizing enzymes, glucose, TP, albumin, and creatinine accompanied by cytopathological disruptions and fibrosis. Although the antioxidant defensive measures were enhanced in the hope to conquer the burden of petroleum contaminants, free radical-mediated peroxidation and cytopathological alterations were still noticeable. These outcome measures could be suggested to decision-making authorities as diagnostic criteria for the early discovery of pollution with volatile hydrophobic compounds during the monitoring programs. On the other side, the inclusion of SP in ration formation reversed the toxicity due to its antioxidant and cytoprotective characteristics. However, the kidney still exhibited histo-architectural lesions even after SP remediation essentially because of insufficient dose and time window. Further studies are strongly warranted to determine the most optimum intervention schedule for SP.

## Data Availability

All data generated or analyzed during this study are included in the research article.
